# Machine-Learning Models of Cognitive Test Performance Incorporating Exposomic Pesticide Biomarkers in Older U.S. Adults

**DOI:** 10.3390/brainsci16080794

**Published:** 2026-07-28

**Authors:** Carlos A. Toro, Giulio Maria Pasinetti

**Affiliations:** 1 Spinal Cord Damage Research Center, James J. Peters Veterans Affairs Medical Center, Bronx, NY 10468, USA; carlos.torochacon@va.gov; 2 VA Sequencing Collaborations United for Research and Epidemiology (seqCURE), James J. Peters Veterans Affairs Medical Center, Bronx, NY 44106, USA; 3 The Bronx Veterans Medical Research Foundation, Bronx, NY 10468, USA; 4 Geriatrics Research, Education and Clinical Center (GRECC), James J. Peters Veterans Affairs Medical Center, Bronx, NY 10468, USA

**Keywords:** cognitive aging, exposome, pesticide biomarkers, DEET, NHANES, LASSO, ridge regression, DSST, CERAD, animal fluency

## Abstract

**Highlights:**

**What are the main findings?**

- Sex, race/ethnicity, and educational attainment were among the strongest predictors of cognitive test performance in NHANES 2011–2014 adults aged ≥60 years with complete cognitive and urinary pesticide biomarker data.
- DEET and desethyl hydroxy-DEET retained negative coefficients for DSST performance in regularized models, particularly among adults aged 60–69 years.

**What are the implications of the main findings?**

- Exposomic pesticide biomarkers may add useful environmental context to demographic, lifestyle, and psychosocial models of cognitive aging.
- Longitudinal and mechanistic studies are needed to determine whether these cross-sectional associations reflect causal pathways, correlated exposures, or residual confounding.

**Abstract:**

**Background/Objectives:** Environmental exposures may contribute to heterogeneity in cognitive aging, yet population-scale datasets integrating exposure biomarkers with cognitive testing remain underused. We evaluated associations between cognitive test performance and demographic, lifestyle, psychosocial, and pesticide-exposure variables in older U.S. adults. **Methods:** Using NHANES 2011–2014 data, we analyzed adults aged ≥60 years with complete cognitive assessments, demographic/lifestyle covariates, Patient Health Questionnaire-9 scores, binge drinking status, and urinary concentrations of eight pesticide biomarkers. Least absolute shrinkage and selection operator (LASSO) and ridge regression models were trained to predict CERAD immediate learning composite scores, Animal Fluency, and Digit Symbol Substitution Test (DSST) performance. Models were evaluated using mean absolute error, mean squared error, root mean squared error, and R-squared. **Results:** The analytic sample included 429 participants. Sex, race/ethnicity, and educational attainment were among the strongest predictors across outcomes. LASSO showed the best overall performance in the full cohort, particularly for DSST (R-squared = 0.4680). DEET and desethyl hydroxy-DEET retained non-zero negative coefficients for DSST, and a reduced model including demographics and these two biomarkers achieved comparable performance (DSST R-squared = 0.475). Age-stratified analyses suggested stronger predictor importance in adults aged 60–69 years. **Conclusions:** These findings support incorporating exposomic biomarkers alongside sociodemographic factors when modeling cognitive test performance in older adults, while emphasizing that cross-sectional NHANES data cannot establish temporality or causality.

## 1. Introduction

According to Centers for Disease Control and Prevention (CDC) data from 2021, life expectancy in the United States was 76.4 years [1]. As the population ages, the burden of age-associated neurodegenerative diseases continues to increase [2]. Alzheimer’s disease (AD) affects an estimated 6.7 million Americans aged ≥65 years [3], and Parkinson’s disease (PD) is projected to affect approximately 1.2 million Americans by 2030 [4]. Both disorders can present with cognitive impairment, and early clinical diagnosis remains challenging [5].

Cognitive impairment is multifactorial and may reflect neurodegenerative pathology, psychiatric symptoms, medication effects, vascular and metabolic comorbidities, alcohol use, and other clinical factors [6,7]. In addition, lifestyle, socioeconomic, educational, and environmental exposures may shape trajectories of cognitive aging and contribute to health inequities [8,9]. Understanding these factors in population-based cohorts may help identify modifiable risks, clarify heterogeneity in cognitive aging, and inform prevention strategies.

### 1.1. Theoretical Background

Cognitive aging is increasingly understood as a life-course process shaped by the interaction of biological vulnerability, clinical comorbidity, lifestyle behaviors, and social determinants of health. Prior population-based studies demonstrate that cognitive test performance in older adults varies by age, educational attainment, sex, and race/ethnicity [9,10]. These variables may reflect differences in cognitive reserve, lifetime access to education and health-promoting resources, occupational exposures, medical comorbidity, and structural inequities that influence both brain health and performance on standardized cognitive assessments. Therefore, environmental exposure analyses in aging populations should be interpreted within a broader biopsychosocial framework rather than as isolated toxicologic associations.

The exposome framework provides a useful theoretical model for this study because it emphasizes the cumulative influence of environmental exposures across the life course. Environmental chemicals, pesticides, diet, alcohol use, occupation, psychosocial stress, and comorbid conditions may interact with aging-related biological vulnerability to influence cognitive trajectories [8]. In this context, pesticide biomarkers are not only indicators of recent chemical exposure but may also serve as measurable components of broader environmental and behavioral exposure patterns. Experimental studies further suggest that selected pesticide-related compounds may influence neuronal vulnerability through cellular stress pathways relevant to neurodegeneration, including stress granule assembly and translational arrest [11].

Pesticides and insect repellents are particularly relevant to cognitive aging research because exposure may occur through residential use, occupational activities, agricultural environments, and vector-control practices. DEET is among the most widely used insect repellents, and desethyl hydroxy-DEET is a urinary metabolite that can be used as a biomarker of recent exposure. Although human epidemiologic evidence linking DEET-related biomarkers to cognition remains limited, experimental findings provide a biologically plausible rationale for hypothesis-generating analyses of DEET-related exposure markers in older adults [11].

The cognitive tests used in NHANES capture complementary domains relevant to aging and neurodegenerative risk. CERAD immediate learning reflects episodic learning and memory processes [12,13,14,15], Animal Fluency assesses semantic retrieval and executive function [16], and the Digit Symbol Substitution Test (DSST) reflects processing speed, sustained attention, and working memory [17]. DSST performance is particularly sensitive to aging and diffuse cognitive dysfunction, making it a useful outcome for evaluating whether environmental biomarkers add predictive context beyond demographic and lifestyle variables [17].

Machine-learning and regularized modeling approaches have been increasingly applied to NHANES and other population-scale datasets to evaluate complex relationships among environmental exposures, clinical characteristics, and health outcomes [18,19,20]. LASSO and ridge regression are well suited for this type of analysis because they can evaluate multiple correlated predictors while reducing overfitting and identifying variables that contribute most strongly to prediction [21]. These methods are not intended to prove causality, but they can help generate testable hypotheses regarding environmental contributors to cognitive aging.

### 1.2. Study Rationale and Objectives

NHANES is a nationally representative U.S. survey that integrates interviews, physical examinations, and laboratory measurements, including biomarkers of environmental exposures [22]. The 2011–2014 cycles are particularly useful for cognitive aging research because they include cognitive testing in older adults together with demographic, psychosocial, lifestyle, and laboratory exposure data [10].

Leveraging NHANES 2011–2014 cognitive testing, we evaluated whether demographic characteristics, depressive symptoms, alcohol use, and urinary biomarkers of selected pesticides are associated with performance on cognitive assessments in older adults. We applied LASSO and ridge regression to model cognitive test scores and to compare the relative contribution of exposomic and non-exposomic predictors. Rather than attempting to diagnose cognitive impairment or infer causality, this analysis was designed as a population-based, hypothesis-generating study to determine whether exposomic pesticide biomarkers contribute to machine-learning models of cognitive performance after accounting for major demographic and behavioral factors.

## 2. Materials and Methods

### 2.1. Data Source and Study Population

The National Health and Nutrition Examination Survey (NHANES) is a continuous, nationally representative program designed to assess the health and nutritional status of the U.S. population [22]. We analyzed NHANES 2011–2014 data, the most recent cycle containing the cognitive assessment battery for older adults, focusing on participants aged ≥60 years.

NHANES 2011–2014 included 2934 participants aged ≥60 years. All NHANES protocols were approved by the National Center for Health Statistics Research Ethics Review Board, and participants provided written informed consent. We applied the following inclusion criteria: (1) complete data for cognitive assessments (CERAD immediate learning, CERAD delayed recall, Animal Fluency [AF], and Digit Symbol Substitution Test [DSST]); (2) complete demographic and lifestyle variables (sex, age, race/ethnicity, education, marital status, military service, and binge drinking); (3) complete Patient Health Questionnaire-9 (PHQ-9) data, summed to a total score ranging from 0 to 27 [23]; and (4) available urinary concentrations for selected pesticide biomarkers. After exclusions for missing data, the analytic sample included 429 participants.

### 2.2. Study Variables and Cognitive Outcomes

Predictor variables were selected a priori from NHANES and included demographics, PHQ-9 total score, binge drinking status, and urinary pesticide biomarkers (Table 1). Outcomes were continuous cognitive test scores: CERAD immediate learning composite (CFDSTSUM), Animal Fluency, and DSST.

Cognitive outcomes included the following: (1) CERAD immediate learning, consisting of three learning trials summed as a composite (CFDSTSUM), and CERAD delayed recall [12,13,14]; (2) the Animal Fluency test, assessing verbal fluency and executive function (score range 1–40) [16]; and (3) the DSST from the WAIS-III, assessing processing speed, sustained attention, and working memory (score range 0–100) [17]. Participants were grouped by age (60–69, 70–79, and ≥80 years). Depressive symptoms were measured using PHQ-9, and binge drinking was defined using CDC criteria (≥5 drinks on an occasion for men or ≥4 drinks for women) [24].

### 2.3. Urinary Pesticide Biomarkers

Urinary pesticide biomarkers (micrograms per liter) included DEET, desethyl hydroxy-DEET, 2,4-D, 4-fluoro-3-phenoxy-benzoic acid, 3-phenoxybenzoic acid, oxypyrimidine, para-nitrophenol, and trans-3-(2,2-dichlorovinyl)-2,2-dimethylcyclopropane carboxylic acid. These biomarkers were analyzed as continuous variables and incorporated into both exploratory association analyses and regularized regression models.

The eight pesticide biomarkers were selected a priori because they were available within the NHANES 2011–2014 cycles that included cognitive testing and provided sufficient completeness for integrated analyses. Additional pesticides, including glyphosate, AMPA, neonicotinoids, and other DEET metabolites, were unavailable or lacked sufficient overlap with cognitive assessment data during these survey cycles.

Urinary pesticide biomarkers were measured by the CDC National Center for Environmental Health using validated analytical methods under standardized NHANES quality assurance procedures. Detailed information regarding analytical precision, accuracy, quality control, recovery, and limits of detection is available in the NHANES Laboratory Procedures Manuals.

### 2.4. Statistical and Machine-Learning Analyses

Continuous variables were summarized as mean +/− SD, and categorical variables as counts and percentages. Pearson correlation and linear regression were used to explore relationships among continuous predictors and cognitive outcomes. For categorical predictors, we assessed normality using the Shapiro–Wilk test and homogeneity of variance using Levene’s test [25,26]. ANOVA was used when assumptions were met; otherwise, the Kruskal–Wallis test or other nonparametric procedures were applied [27].

**Figure 1:**
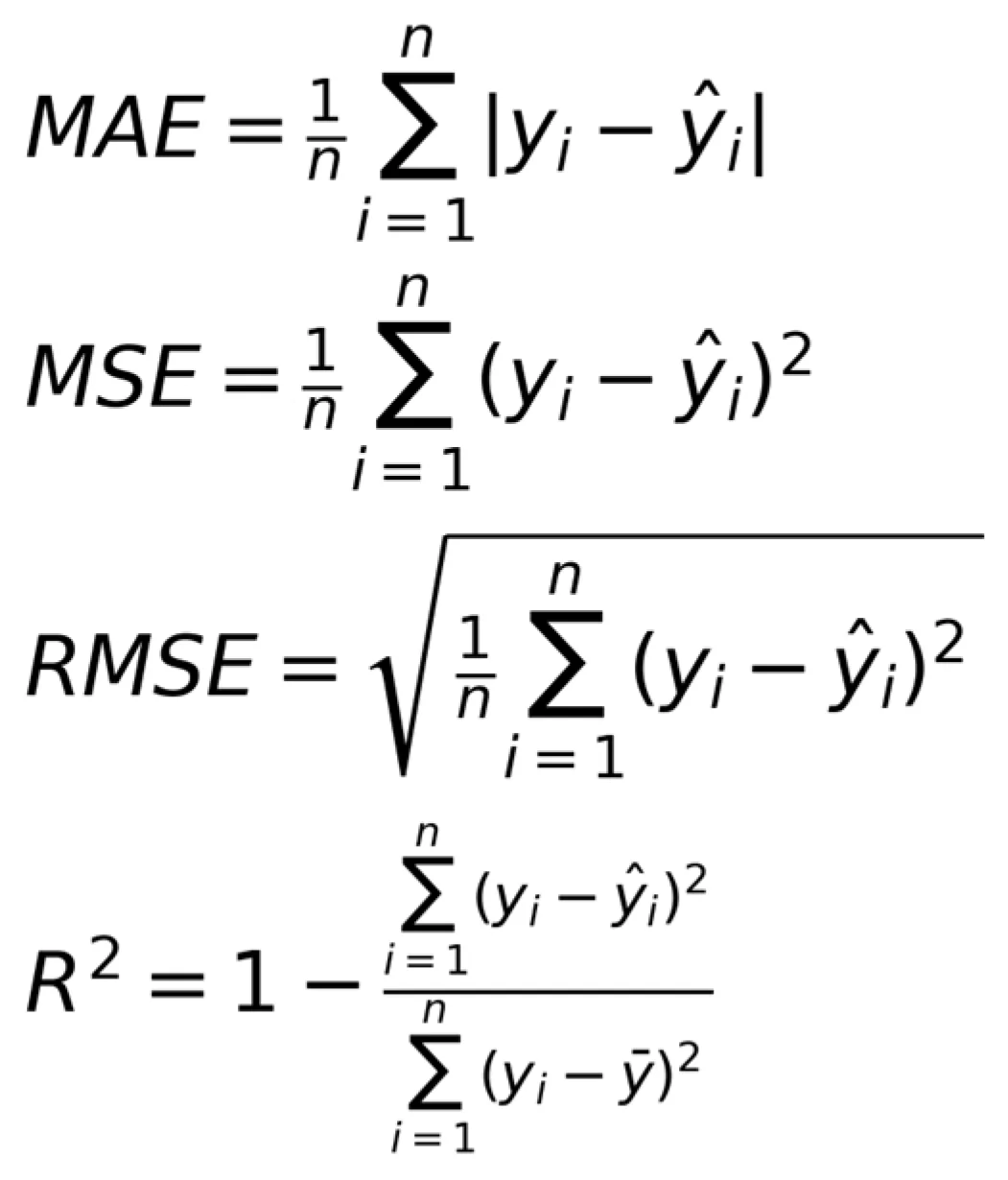
Model-performance metrics used for the test cohort. y_i represents observed cognitive test performance, y-hat_i represents predicted performance, y-bar represents the observed mean, and n represents the number of observations. All analyses were completed using R (v4.3.1).

The dataset was split into training and test sets (80:20). Using the training data, we fit LASSO and ridge regression models to predict cognitive test performance. Modeling and evaluation were implemented in R using the glmnet and caret packages. LASSO applies an L1 penalty that can shrink some coefficients to zero, enabling feature selection, whereas ridge regression applies an L2 penalty that shrinks coefficients without eliminating predictors [21]. We used 10-fold cross-validation to select the regularization parameter lambda and evaluated the lambda-minimum solution. Variable importance was assessed using coefficient magnitudes. To examine potential age heterogeneity, we repeated the modeling procedures within three age strata (60–69, 70–79, and ≥80 years). Model performance on the held-out test set was quantified using mean absolute error, mean squared error, root mean squared error, and R-squared (Figure 1) [28].

Although raw urinary pesticide concentrations are reported in the descriptive statistics to preserve their original units and facilitate interpretation, continuous predictors were automatically standardized (mean-centered and scaled to unit variance) during model fitting by the glmnet package. Consequently, coefficient estimation and variable selection were performed using standardized predictors.

## 3. Results

### 3.1. Cohort Characteristics and Preliminary Associations

After applying inclusion criteria, 429 participants remained in the analytic cohort. Based on prior NHANES analyses [10], CERAD delayed recall did not show a clear age-associated trend in this dataset. Accordingly, subsequent analyses focused on three outcomes: CERAD immediate learning composite (CFDSTSUM), Animal Fluency, and DSST.

We evaluated associations between demographic/lifestyle variables and cognitive outcomes. Age and PHQ-9 score showed modest negative correlations with CFDSTSUM, AF, and DSST (Table 2). For categorical predictors, differences in cognitive scores were assessed using ANOVA or Kruskal–Wallis tests as appropriate. Sex, race/ethnicity, and education were associated with CFDSTSUM and DSST (*p* < 0.05), and race/ethnicity and education were associated with AF (*p* < 0.05). Marital status was associated with AF only (*p* < 0.05), and binge drinking was associated with DSST (*p* < 0.05).

### 3.2. Pesticide Biomarker Associations

**Figure 2:**
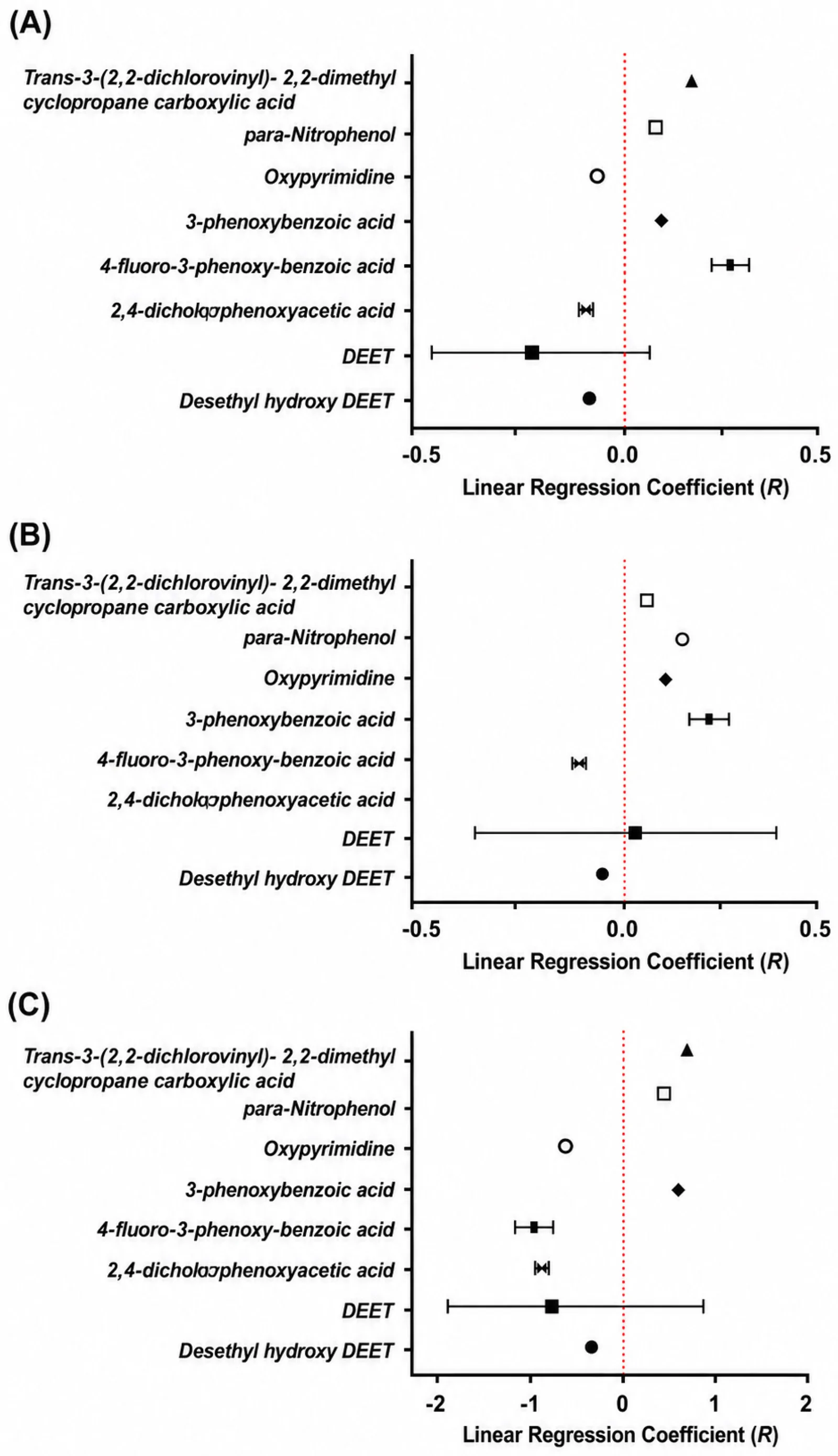
Individual linear regression analysis between pesticide concentrations and cognitive performance. (**A**) Sum of CERAD immediate recall assessments versus pesticide concentrations; (**B**) Animal Fluency test versus pesticide concentrations; (**C**) Digit Symbol Substitution Test versus pesticide concentrations.

Linear regression analyses of urinary pesticide biomarkers suggested that trans-3-(2,2-dichlorovinyl)-2,2-dimethylcyclopropane carboxylic acid was associated with CERAD immediate learning composite performance (*p* < 0.05; Figure 2).

**Figure 3:**
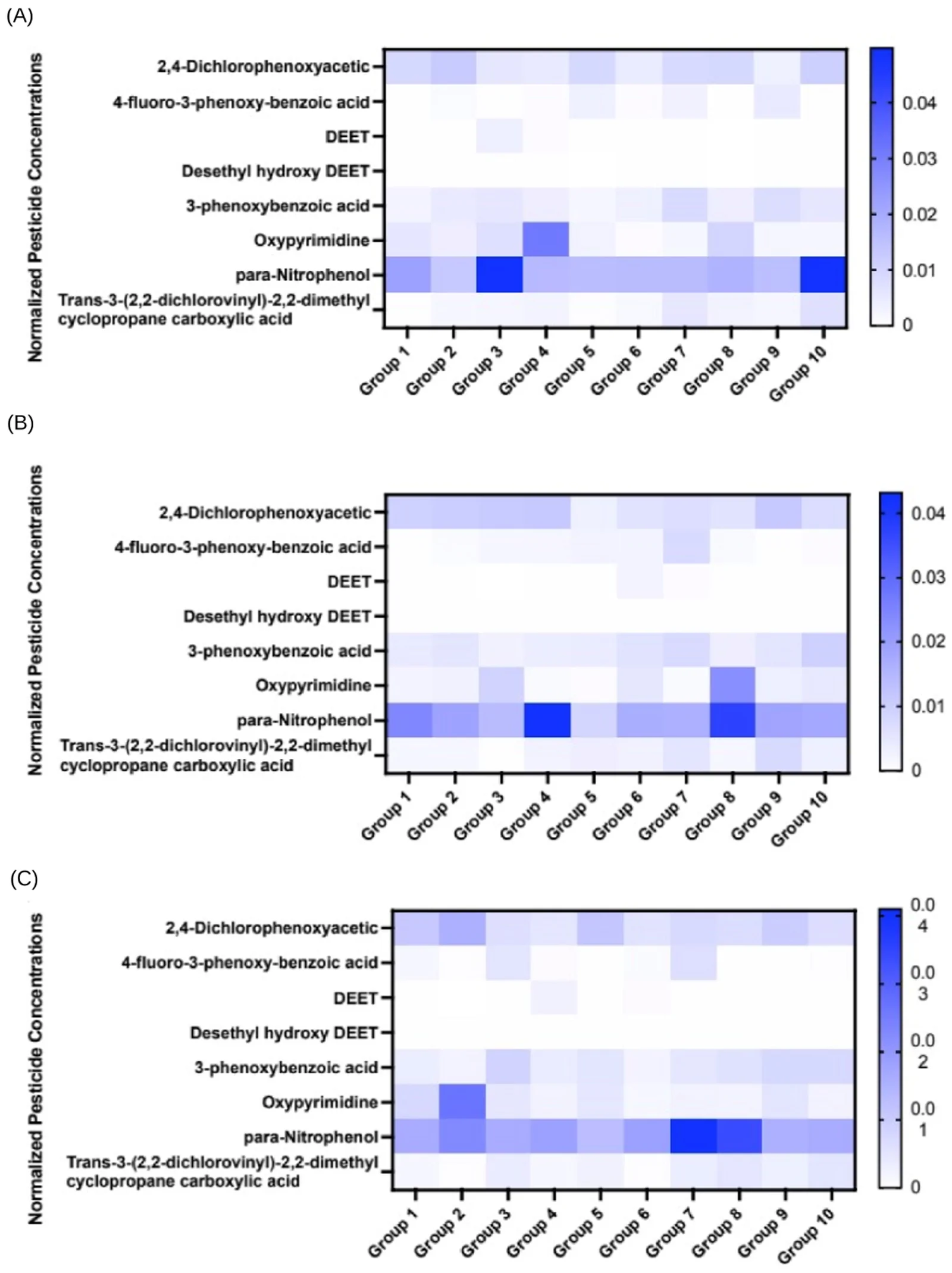
Normalized pesticide concentrations by cognitive performance ranked groups. Groups were divided into 10 groups based on average scores on each assessment. (**A**) Sum of CERAD immediate recall assessments versus pesticide concentrations; (**B**) Animal Fluency test versus pesticide concentrations; (**C**) Digit Symbol Substitution Test versus pesticide concentrations.

We stratified participants into deciles by cognitive test performance and visualized normalized urinary pesticide concentrations (Figure 3). Concentrations varied across performance deciles; trans-3-(2,2-dichlorovinyl)-2,2-dimethylcyclopropane carboxylic acid tended to be higher in higher-scoring groups, whereas other pesticide biomarkers did not show consistent monotonic patterns.

### 3.3. Model Performance and Variable Importance

**Figure 4:**
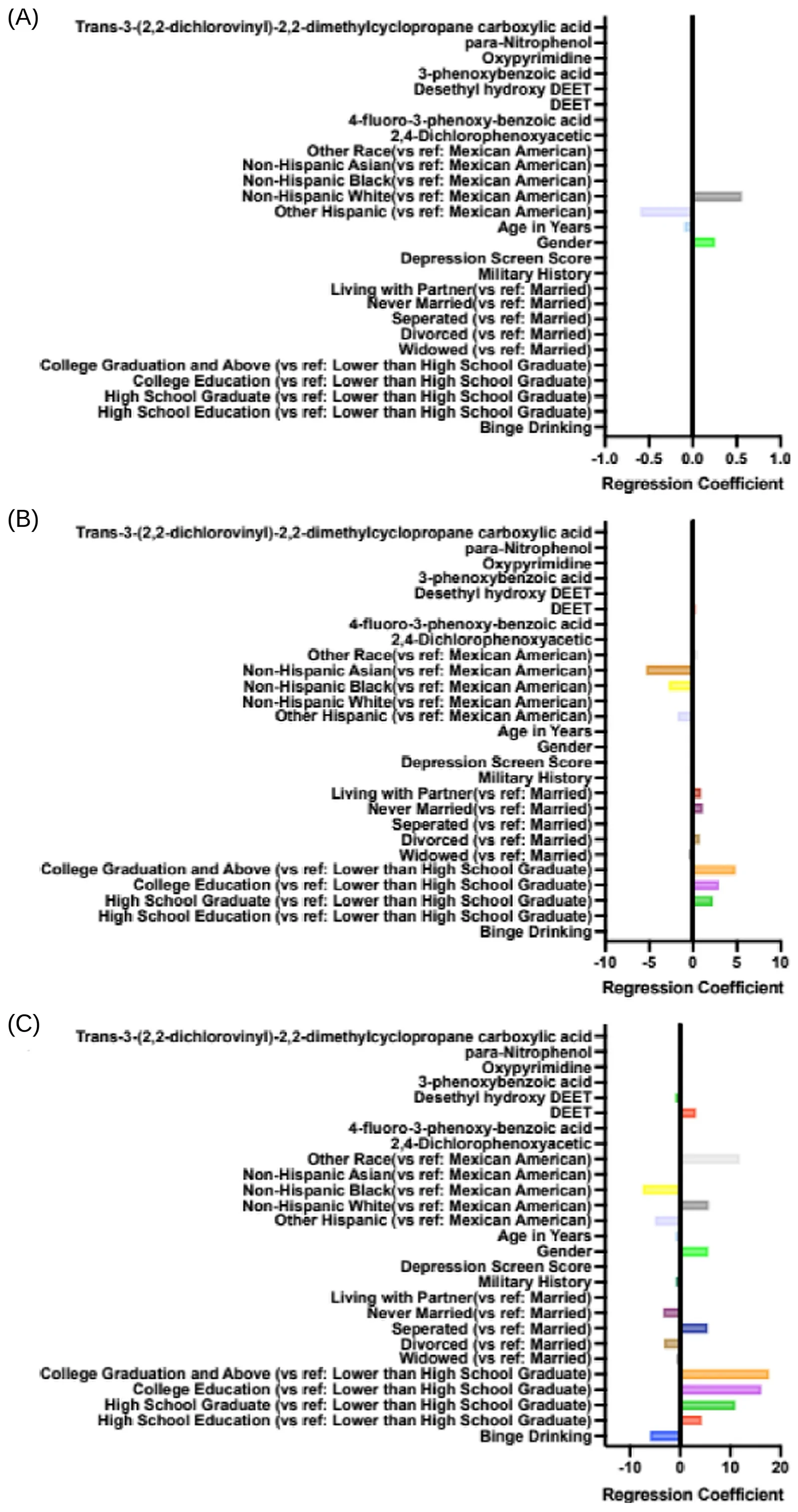
DEET and desethyl hydroxy-DEET retained non-zero coefficients in LASSO models of cognitive test performance, including DSST performance. Based on coefficient magnitudes (**A**) Sex; (**B**) race/ethnicity, and (**C**) education which were the most influential predictors.

We used 10-fold cross-validation to train two regularized regression models (LASSO and ridge) for each cognitive outcome. In the full cohort, LASSO showed better overall performance (MAE: 3.5588 [CFDSTSUM], 10.3564 [DSST], 4.6006 [AF]; MSE: 21.6631 [CFDSTSUM], 160.9348 [DSST], 34.5648 [AF]; RMSE: 4.6544 [CFDSTSUM], 12.6860 [DSST], 5.8792 [AF]; R-squared: 0.1444 [CFDSTSUM], 0.4680 [DSST], 0.1158 [AF]). Based on coefficient magnitudes, sex, race/ethnicity, and education were consistently among the most influential predictors. DEET and desethyl hydroxy-DEET also had non-zero coefficients for DSST (Figure 4). We therefore fit a parsimonious LASSO model retaining all demographics and the two DEET-related biomarkers.

**Figure 5:**
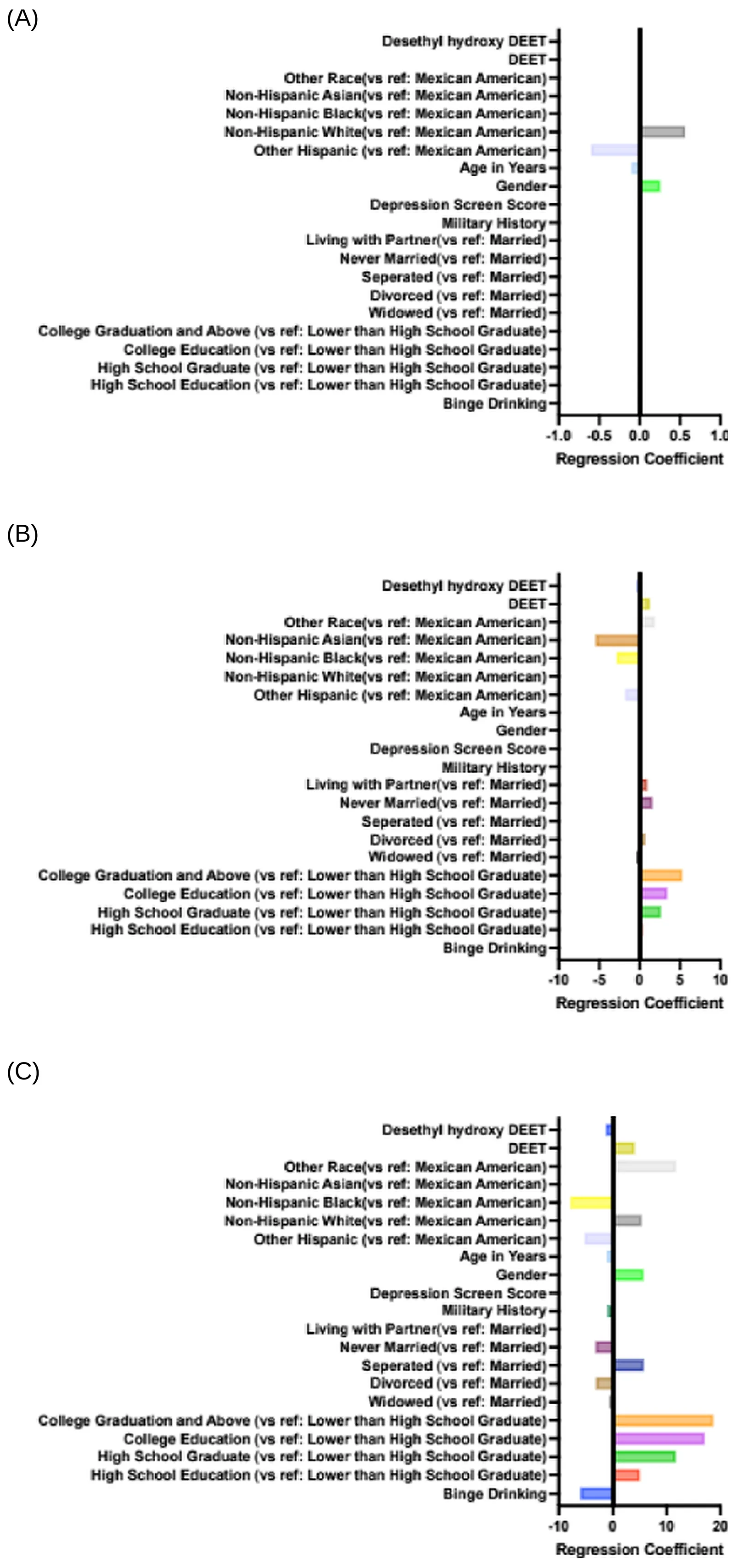
Reduced LASSO model (demographics plus DEET biomarkers) achieved comparable DSST predictive performance. Based on coefficient magnitudes (**A**) Sex; (**B**) race/ethnicity, and (**C**) education which were the most influential predictors.

In the reduced model (demographics plus DEET and desethyl hydroxy-DEET), performance was similar to or slightly improved relative to the full model (DSST R-squared = 0.475; Figure 5). Most predictors retained comparable relative importance, and the absolute magnitude of coefficients increased for the majority of variables (83%). These results suggest that DEET-related biomarkers may contribute incremental explanatory power for DSST beyond demographic factors.

### 3.4. Age-Stratified Analyses

**Figure 6:**
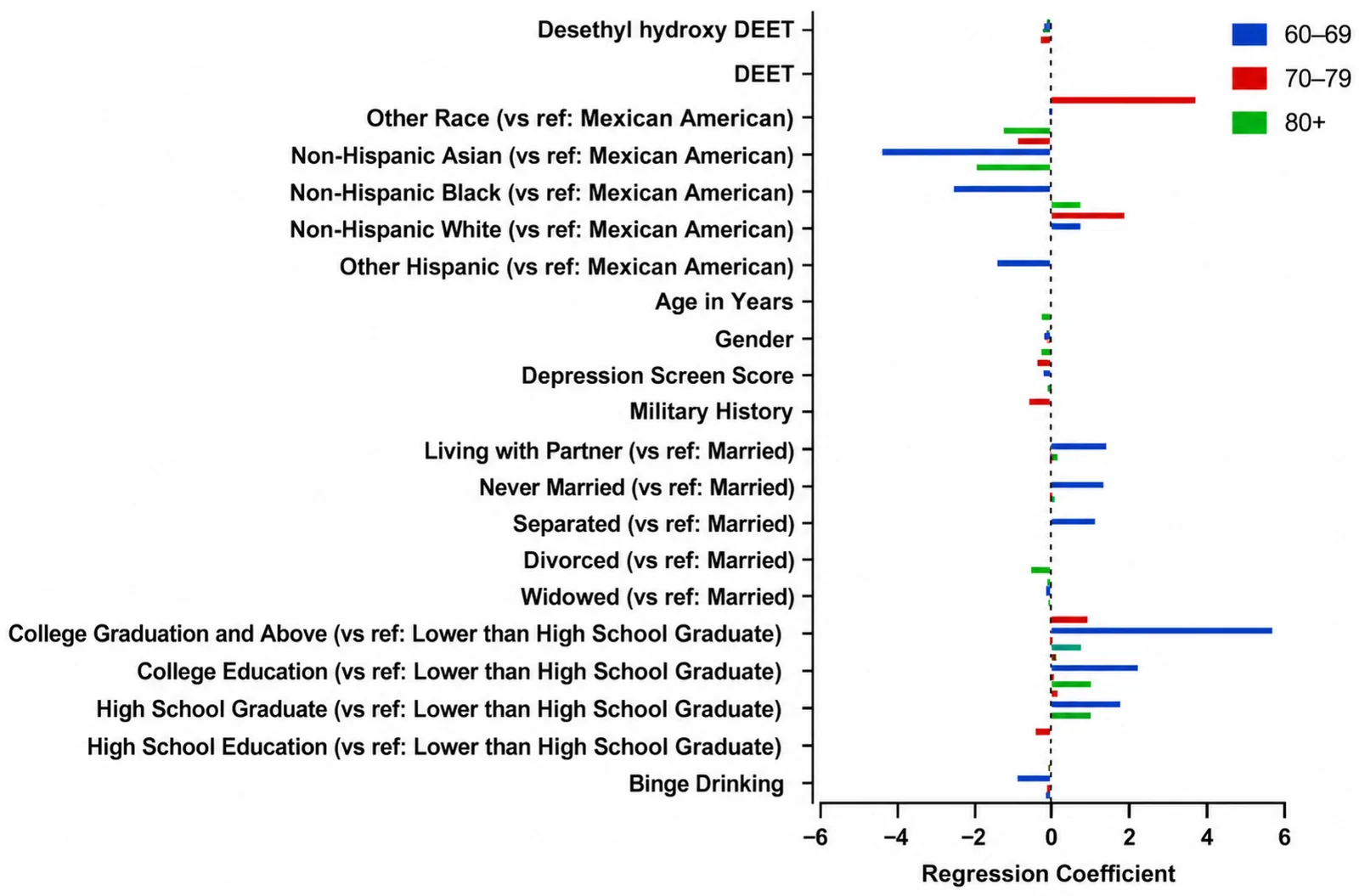
LASSO variable importance stratified by age group.

Age-stratified analyses indicated that model performance was highest in the 60–69-year subgroup (R-squared: 0.0773 [CFDSTSUM], 0.3143 [DSST], 0.2590 [AF]), with lower performance in older strata. Variable importance was generally larger in the 60–69-year group, consistent with greater between-person variability explained by the included predictors in this subgroup (Figure 6).

## 4. Discussion

In this study, we used NHANES 2011–2014 data to examine whether urinary pesticide biomarkers, together with demographic, lifestyle, and psychosocial variables, were associated with cognitive test performance in older U.S. adults. The main findings were that sex, race/ethnicity, and educational attainment were among the strongest predictors across cognitive outcomes, while DEET and desethyl hydroxy-DEET retained negative coefficients for DSST performance in regularized models. LASSO showed the best overall performance in the full cohort, particularly for DSST, supporting the concept that cognitive performance in older adults is shaped by a combination of sociodemographic, behavioral, psychosocial, and environmental factors.

The strong contribution of education, sex, and race/ethnicity is consistent with prior literature showing that cognitive test performance reflects not only neurobiological aging but also lifelong social, educational, and health-related exposures [9,10]. In the present cohort, these variables were associated with several cognitive outcomes, including CERAD immediate learning, DSST, and Animal Fluency. These findings emphasize that exposomic analyses must account for major social and demographic determinants of cognitive performance. Educational attainment may partly reflect cognitive reserve, while race/ethnicity may capture the influence of structural, social, occupational, environmental, and health-care-related factors that are not fully measured in NHANES. Therefore, the observed pesticide-related associations should be interpreted as occurring within a broader context of cumulative life-course exposures.

The finding that DEET-related biomarkers retained negative coefficients for DSST is noteworthy because DSST is sensitive to processing speed, sustained attention, and working memory [17]. In the reduced model, demographics plus DEET and desethyl hydroxy-DEET achieved comparable DSST predictive performance relative to the full model. Although the magnitude and interpretation of these associations require caution, the results are biologically plausible in light of experimental studies suggesting that DEET can induce cellular stress responses relevant to neuronal vulnerability [11]. The findings do not indicate that DEET exposure causes cognitive decline, but they suggest that DEET-related biomarkers may capture environmental or behavioral exposure patterns associated with cognitive performance.

In addition to inducing cellular stress responses, DEET has been reported in experimental studies to exhibit acetylcholinesterase inhibitory activity, particularly under conditions of higher exposure or in combination with other neurotoxic compounds. Although environmental exposure levels in humans are substantially lower than those used in many experimental models, disruption of cholinergic neurotransmission represents another biologically plausible mechanism through which DEET-related exposure could influence cognitive processes such as attention, processing speed, and working memory.

Age-stratified analyses suggested that model performance and predictor importance were strongest among adults aged 60–69 years, with lower performance in older age strata. One possible interpretation is that early older adulthood may represent a period in which interindividual variability in cognitive performance is more strongly influenced by modifiable or measurable exposures, whereas in later decades cognitive performance may be increasingly shaped by survival effects, neurodegenerative disease burden, frailty, comorbidities, or unmeasured clinical factors. This finding should be considered exploratory but may help guide future studies focused on earlier windows of cognitive aging.

Although LASSO demonstrated slightly better predictive performance in this dataset and produced more interpretable sparse models, ridge regression yielded comparable prediction accuracy while retaining correlated predictors. Because highly correlated exposures frequently coexist in environmental epidemiology, ridge regression may better preserve distributed exposure effects, whereas LASSO favors parsimonious variable selection. Accordingly, variables selected by LASSO should be interpreted as predictive rather than definitive causal determinants.

### 4.1. Limitations

This study has several important limitations. First, NHANES is cross-sectional; therefore, the temporal sequence between pesticide biomarker levels and cognitive test performance cannot be established. Urinary pesticide biomarkers generally reflect recent exposure and may not represent cumulative, occupational, residential, or lifetime exposure history. As a result, the observed associations may reflect recent use patterns, correlated environmental exposures, or behavioral factors rather than direct neurotoxic effects.

Second, the analytic sample was reduced substantially by the requirement for complete cognitive, demographic, lifestyle, PHQ-9, and urinary pesticide biomarker data. Complete-case filtering may introduce selection bias if participants with missing biomarker or cognitive data differed systematically from those included in the analysis. The final sample size also limited statistical power, particularly for age-stratified analyses and for detecting modest exposure-cognition associations.

Third, the analysis did not fully account for all potential confounders. Important factors such as occupational history, residential pesticide exposure, diet, comorbid vascular and metabolic disease, medication use, sleep, physical activity, genetic risk, and detailed socioeconomic indicators were not comprehensively modeled. In addition, urinary pesticide concentrations were not adjusted for urinary dilution, such as creatinine or specific gravity, which may influence biomarker interpretation. The observed associations may also reflect shared determinants of pesticide exposure and cognitive performance, including occupational status, residential environment, metabolic health, medication use affecting xenobiotic metabolism, dietary patterns, physical activity, and other socioeconomic or behavioral factors that were incompletely captured in NHANES. Consequently, the present findings should be interpreted as hypothesis-generating rather than evidence of direct toxicological effects.

Fourth, the cognitive outcomes reflect test performance rather than clinical diagnosis. CERAD immediate learning, Animal Fluency, and DSST are useful measures of cognitive domains relevant to aging, but they cannot determine whether a participant has mild cognitive impairment, Alzheimer’s disease, Parkinson’s disease, or another neurocognitive disorder. Therefore, the findings should be interpreted as associations with cognitive performance, not with disease status.

Finally, the machine-learning models were designed for hypothesis generation rather than clinical prediction. Although LASSO and ridge regression help manage correlated predictors and reduce overfitting, model performance was modest for some outcomes, especially CERAD immediate learning and Animal Fluency. External validation in independent cohorts is needed before these models can be considered generalizable. Importantly, exclusion was based on incomplete biomarker data rather than nondetectable pesticide concentrations. NHANES routinely reports measurements below the analytical limit of detection using standard substitution procedures, allowing low-exposure individuals to remain represented. Nevertheless, complete-case analysis may still introduce selection bias if participants missing laboratory measurements differed systematically from those included.

### 4.2. Practical Implications

Despite these limitations, the study has several practical implications. First, the findings support the value of integrating exposomic biomarkers into studies of cognitive aging. Demographic and educational variables remained dominant predictors, but pesticide biomarkers may add environmental context that is not captured by traditional clinical or sociodemographic measures. This is particularly relevant for population health research because environmental exposures are potentially modifiable and may contribute to disparities in cognitive aging.

Second, the results highlight DSST as a potentially sensitive cognitive outcome for environmental exposure research in older adults. DSST performance may capture subtle changes in processing speed, attention, and working memory that are influenced by both aging and environmental vulnerability. Future epidemiologic studies evaluating pesticides or other environmental biomarkers may benefit from including DSST or comparable processing-speed measures.

Third, the findings suggest that environmental exposure assessment should be interpreted alongside social determinants of health. Race/ethnicity and education were strong predictors in the models, indicating that environmental risk cannot be separated from broader social and structural factors. Public health approaches aimed at preserving cognitive health in aging populations should therefore consider combined strategies that address environmental exposure reduction, health equity, education, lifestyle, and access to preventive care.

Fourth, although the study does not support individual-level clinical decision-making, it provides a rationale for more detailed exposure histories in research settings. For older adults with occupational, agricultural, military, or residential pesticide exposure histories, future studies may consider combining questionnaire-based exposure assessment with biomarker data and longitudinal cognitive follow-up.

### 4.3. Future Studies

Future studies should use longitudinal designs to determine whether pesticide biomarkers predict cognitive decline over time. Repeated biomarker measurements would help distinguish transient exposure from persistent or cumulative exposure patterns. Longitudinal cognitive testing would also allow investigators to determine whether DEET-related biomarkers are associated with change in DSST or other cognitive domains rather than only cross-sectional performance.

Future analyses should also incorporate broader exposomic data, including additional pesticides, metals, air pollution markers, persistent organic pollutants, diet-related biomarkers, and occupational or residential exposure histories. Because individuals are exposed to mixtures rather than single compounds, mixture-based methods may better characterize the combined effects of environmental chemicals on cognitive aging.

Mechanistic studies are also needed to clarify whether DEET or related compounds influence pathways relevant to neurodegeneration. Experimental models could examine oxidative stress, mitochondrial dysfunction, neuroinflammation, synaptic function, proteostasis, and stress granule biology in neuronal and glial systems. These studies would help determine whether the epidemiologic associations observed here reflect plausible biological effects or indirect exposure patterns.

Finally, future work should validate these findings in independent cohorts with larger sample sizes, more diverse participants, and richer clinical characterization. Studies that combine exposomic biomarkers with neuroimaging, blood-based neurodegeneration biomarkers, genomics, transcriptomics, and detailed neuropsychological testing may help identify subgroups of older adults who are more vulnerable to environmental exposures. Such work could eventually inform prevention strategies aimed at reducing modifiable environmental contributors to cognitive decline.

## 5. Conclusions

Regularized regression models applied to NHANES 2011–2014 data highlight that demographic factors, particularly sex, race/ethnicity, and educational attainment, are strong correlates of cognitive test performance in older adults. The results also suggest that DEET-related pesticide biomarkers may be associated with DSST performance and may add environmental context to models of cognitive aging. Together, these findings support the incorporation of exposomic biomarkers into cognitive aging research while emphasizing that the present results are hypothesis-generating. Longitudinal, mechanistic, and externally validated studies are needed to determine whether DEET-related biomarkers and other pesticide exposures contribute causally to cognitive decline or instead reflect broader environmental, behavioral, and social exposure patterns.

## Figures and Tables

**Table 1:** Baseline characteristics of the filtered study cohort (N = 429).

Characteristics	N (%)/Mean ± SD
Gender	
Male	238 (55.48%)
Female	191 (44.52%)
Age	69.23 ± 6.91
**Race**	
Mexican American	35 (8.16%)
Other Hispanic	33 (7.69%)
Non-Hispanic White	231 (53.85%)
Non-Hispanic Black	91 (21.21%)
Non-Hispanic Asian	35 (8.16%)
Other Race—Including Multi-Racial	4 (0.93%)
**Highest educational level**	
Less than 9th grade	36 (8.39%)
9–11th grade (Includes 12th grade with no diploma)	39 (9.09%)
High school graduate/GED or equivalent	87 (20.28%)
Some college or AA degree	132 (30.77%)
College graduate or above	135 (31.47%)
**Marital status**	
Married	253 (58.97%)
Widowed	71 (16.55%)
Divorced	57 (13.29%)
Separated	11 (2.56%)
Never married	24 (5.59%)
Living with partner	13 (3.03%)
**Military Status**	
Yes	97 (22.61%)
No	332 (77.38%)
Depression screen score	2.965 ± 4.13
**Binge drinking**	
Yes	18 (4.20%)
No	411 (95.80%)
DEET (μg/L)	0.1119 ± 0.77
Desethyl hydroxyDEET (μg/L)	0.3506 ± 2.69
2,4-D (μg/L)	0.6364 ± 1.40
4-fluoro-3-phenoxy-benzoic acid (μg/L)	0.1647 ± 0.62
3-phenoxybenzoic acid (μg/L)	2.173 ± 5.24
oxypyrimidine (μg/L)	0.3109 ± 1.98
para-nitrophenol (μg/L)	1.370 ± 4.26
trans-3-(2,2-dichlorovinyl)-2,2-dimethylcyclopropane carboxylic acid (μg/L)	1.732 ± 5.89
CERAD immediate learning	19.19 ± 4.47
CERAD delayed recall	6.152 ± 2.19
Animal Fluency (AF) Test	17.6 ± 5.63
Digit Symbol Substitution (DSST) Test	49.08 ± 17.63

**Table 2:** Comparison of demographic factors and cognitive test performance.

Patient Demographics	Cognitive Health Status	Statistical Method	*p* Value/r *
Gender	CFDSTSUM	Kruskal–Wallis test	<0.05
	DSST	ANOVA	<0.05
	AF	Kruskal–Wallis test	0.6111
Age	CFDSTSUM	Pearson	−0.28540460 *
	DSST	Pearson	−0.3238759 *
	AF	Pearson	−0.2147906 *
Race	CFDSTSUM	Kruskal–Wallis test	<0.05
	DSST	ANOVA	<0.05
	AF	Kruskal–Wallis test	<0.05
Highest educational level	CFDSTSUM	Kruskal–Wallis test	<0.05
	DSST	ANOVA	<0.05
	AF	Kruskal–Wallis test	<0.05
Marital status	CFDSTSUM	Kruskal–Wallis test	0.4946
	DSST	Kruskal–Wallis test	0.547
	AF	Kruskal–Wallis test	<0.05
Military Status	CFDSTSUM	Kruskal–Wallis test	0.4005
	DSST	Kruskal–Wallis test	0.1653
	AF	Kruskal–Wallis test	0.9113
Depression screen score	CFDSTSUM	Pearson	−0.05422451 *
	DSST	Pearson	−0.1417372 *
	AF	Pearson	−0.1190295 *
Binge drinking	CFDSTSUM	Kruskal–Wallis test	0.06564
	DSST	ANOVA	<0.05
	AF	Kruskal–Wallis test	0.4741

* For continuous variables (Pearson), the value shown is the correlation coefficient (r).

## Data Availability

Publicly available NHANES 2011–2014 datasets were analyzed in this study and are available from the CDC/National Center for Health Statistics NHANES website.

## Abbreviations

AD	Alzheimer’s disease
AF	Animal Fluency
CDC	Centers for Disease Control and Prevention
CERAD	Consortium to Establish a Registry for Alzheimer’s Disease
CFDSTSUM	CERAD immediate learning composite score
DEET	N,N-diethyl-meta-toluamide
DSST	Digit Symbol Substitution Test
LASSO	Least absolute shrinkage and selection operator
MAE	Mean absolute error
MSE	Mean squared error
NHANES	National Health and Nutrition Examination Survey
PD	Parkinson’s disease
PHQ-9	Patient Health Questionnaire-9
RMSE	Root mean squared error
WAIS-III	Wechsler Adult Intelligence Scale, Third Edition

## References

[1] Centers for Disease Control and Prevention Mortality in the United States, 2021. https://www.cdc.gov/nchs/products/databriefs/db456.htm.

[2] Kochanek K.D., Murphy S.L., Xu J.Q., Arias E. (2023). Deaths: Final Data for 2020. Natl. Vital Stat. Rep..

[3] Alzheimer’s Association (2023). 2023 Alzheimer’s disease facts and figures. Alzheimers Dement..

[4] Parkinson’s Foundation Statistics. https://www.parkinson.org/understanding-parkinsons/statistics.

[5] Xu Y., Yan J., Zhou P., Li J., Gao H., Xia Y., Wang Q. (2012). Neurotransmitter receptors and cognitive dysfunction in Alzheimer’s disease and Parkinson’s disease. Prog. Neurobiol..

[6] Langa K.M., Levine D.A. (2014). The diagnosis and management of mild cognitive impairment: A clinical review. JAMA.

[7] Rao R., Creese B., Aarsland D., Kalafatis C., Khan Z., Corbett A., Ballard C. (2022). Risky drinking and cognitive impairment in community residents aged 50 and over. Aging Ment. Health.

[8] Dominguez L.J., Veronese N., Vernuccio L., Catanese G., Inzerillo F., Salemi G., Barbagallo M. (2021). Nutrition, physical activity, and other lifestyle factors in the prevention of cognitive decline and dementia. Nutrients.

[9] Hale J.M., Schneider D.C., Mehta N.K., Myrskyla M. (2020). Cognitive impairment in the U.S.: Lifetime risk, age at onset, and years impaired. SSM Popul. Health.

[10] Brody D.J., Kramarow E.A., Taylor C.A., McGuire L.C. (2019). Cognitive performance in adults aged 60 and over: National Health and Nutrition Examination Survey, 2011–2014. Natl. Health Stat. Rep..

[11] Bhadauriya P., Parihar R., Ganesh S. (2021). Pesticides DEET, fipronil and maneb induce stress granule assembly and translation arrest in neuronal cells. Biochem. Biophys. Rep..

[12] Morris J.C., Heyman A., Mohs R.C., Hughes J.P., van Belle G., Fillenbaum G., Mellits E.D., Clark C. (1989). The Consortium to Establish a Registry for Alzheimer’s Disease (CERAD). Part I. Clinical and neuropsychological assessment of Alzheimer’s disease. Neurology.

[13] Fillenbaum G.G., van Belle G., Morris J.C., Mohs R.C., Mirra S.S., Davis P.C., Tariot P.N., Silverman J.M., Clark C.M., Welsh-Bohmer K.A. (2008). Consortium to Establish a Registry for Alzheimer’s Disease (CERAD): The first twenty years. Alzheimers Dement..

[14] Sotaniemi M., Pulliainen V., Hokkanen L., Pirttilä T., Hallikainen I., Soininen H., Hänninen T. (2012). CERAD-neuropsychological battery in screening mild Alzheimer’s disease. Acta Neurol. Scand..

[15] Chandler M.J., Lacritz L.H., Hynan L.S., Barnard H.D., Allen G., Deschner M., Weiner M.F., Cullum C.M. (2005). A total score for the CERAD neuropsychological battery. Neurology.

[16] Rofes A., de Aguiar V., Jonkers R., Oh S.J., DeDe G., Sung J.E. (2020). What drives task performance during Animal Fluency in people with Alzheimer’s disease?. Front. Psychol..

[17] Jaeger J. (2018). Digit Symbol Substitution Test: The case for sensitivity over specificity in neuropsychological testing. J. Clin. Psychopharmacol..

[18] Li X., Zhao Y., Zhang D., Kuang L., Huang H., Chen W., Fu X., Wu Y., Li T., Zhang J. (2023). Development of an interpretable machine learning model associated with heavy metals’ exposure to identify coronary heart disease among US adults via SHAP: Findings of the US NHANES from 2003 to 2018. Chemosphere.

[19] Wei H., Sun J., Shan W., Xiao W., Wang B., Ma X., Hu W., Wang X., Xia Y. (2022). Environmental chemical exposure dynamics and machine learning-based prediction of diabetes mellitus. Sci. Total Environ..

[20] Li W., Zeng L., Yuan S., Shang Y., Zhuang W., Chen Z., Lyu J. (2023). Machine learning for the prediction of cognitive impairment in older adults. Front. Neurosci..

[21] Liang L., Rasmussen M.H., Piening B., Shen X., Chen S., Röst H., Snyder J.K., Tibshirani R., Skotte L., Lee N.C. (2020). Metabolic dynamics and prediction of gestational age and time to delivery in pregnant women. Cell.

[22] Centers for Disease Control and Prevention About the National Health and Nutrition Examination Survey. https://www.cdc.gov/nchs/nhanes/about_nhanes.htm.

[23] Kroenke K., Spitzer R.L., Williams J.B.W. (2001). The PHQ-9: Validity of a brief depression severity measure. J. Gen. Intern. Med..

[24] Centers for Disease Control and Prevention Binge Drinking. https://www.cdc.gov/alcohol/excessive-drinking-data/index.html.

[25] Yap B.W., Sim C.H. (2011). Comparisons of various types of normality tests. J. Stat. Comput. Simul..

[26] Sharma D., Kibria B.M.G. (2013). On some test statistics for testing homogeneity of variances: A comparative study. J. Stat. Comput. Simul..

[27] Nahm F.S. (2016). Nonparametric statistical tests for the continuous data: The basic concept and the practical use. Korean J. Anesthesiol..

[28] Azarafza M., Hajialilue Bonab M., Derakhshani R. (2022). A deep learning method for the prediction of the index mechanical properties and strength parameters of marlstone. Materials.

